# Optimization of Gel Flooding during the High Water Cut Stage in a Conglomerate Reservoir of the Xinjiang A Oilfield

**DOI:** 10.3390/polym15071809

**Published:** 2023-04-06

**Authors:** Xiankang Xin, Qian Liu, Saijun Liu, Gaoming Yu, Qingshan Wan

**Affiliations:** 1 School of Petroleum Engineering, Yangtze University, Wuhan 430100, China; 2 Hubei Key Laboratory of Oil and Gas Drilling and Production Engineering, Yangtze University, Wuhan 430100, China; 3 School of Petroleum Engineering, National Engineering Research Center for Oil & Gas Drilling and Completion Technology, Yangtze University, Wuhan 430100, China; 4 Research Institute of Experimental Detection, Xinjiang Oilfield Branch of PetroChina, Karamay 834000, China

**Keywords:** conglomerate reservoir, high water cut stage, gel flooding, numerical simulation, optimization

## Abstract

Influenced by water injection, a dominant flow channel is easily formed in the high water cut stage of a conglomerate reservoir, resulting in the inefficient or ineffective circulation of the injected water. With gel flooding as one of the effective development methods to solve the above problems, its parameter optimization determines its final development effect, which still faces great challenges. A new optimization method for gel flooding is proposed in this paper. Firstly, the gel flooding parameters were obtained through physical experiments; then, an experimental model of gel flooding was established according to the target reservoir, and parameter sensitivity analysis was carried out. Next, a history matching of the gel flooding experiment was carried out. Finally, history matching of the target reservoir was also carried out, and a gel flooding scheme was designed and optimized to determine the best parameters. The experimental results showed that the gelation time was 4 h and the gel viscosity was 6332 mPa·s; the breakthrough pressure, resistance factor (RF), and residual resistance factor (RRF) all decreased with the increase in permeability. The gel had a good profile control ability and improved oil recovery by 16.40%. The numerical simulation results illustrated that the porosity of the high permeability layer (HPL) had the greatest impact on the cumulative oil production (COP) of the HPL, and the maximum polymer adsorption value of the HPL had the largest influence on the COP of the low permeability layer (LPL) and the water cut of both layers. Benefiting from parameter sensitivity analysis, history matching of the gel flooding experiment and a conglomerate reservoir in the Xinjiang A Oilfield with less time consumed and good quality was obtained. The optimization results of gel flooding during the high water cut stage in a conglomerate reservoir of the Xinjiang A Oilfield were as follows: the gel injection volume, injection rate, and polymer concentration were 2000 m^3^, 50 m^3^/d, and 2500 mg/L, respectively. It was predicted that the water cut would decrease by 6.90% and the oil recovery would increase by 2.44% in two years. This paper not only provides a more scientific and efficient optimization method for gel flooding in conglomerate reservoirs but also has important significance for improving the oil recovery of conglomerate reservoirs.

## 1. Introduction

The opening-up of China will inevitably bring about rapid economic development, and the demand for oil and gas will also grow. In the face of the growing demand for oil and gas, it is more important to develop domestic oil and gas resources in addition to relying on foreign imports. In 2022, China’s external dependence on crude oil was 70.9%, down from 72% in 2021, but still far beyond the 50% warning line, so there is an urgent need to increase domestic crude oil development [1,2]. The development of conventional oil reservoirs in China has reached a very high level and scale, and it is difficult to improve its development to a higher level for a long time into the future. Therefore, some special oil reservoirs have begun to enter the view of crude oil developers or companies. Among them, conglomerate oil reservoirs have attracted great attention because of their rich geological reserves and large development potential [3,4,5,6]. However, conglomerate reservoirs are a special kind of lithologic reservoir, and they still present problems worldwide due to their complex and variable lithology, the complex modal characteristics of pores, poor reservoir physical properties, and strong heterogeneity [7,8,9,10]. Water flooding is one of the common development methods for conglomerate reservoirs, but long-term water flooding can form a high permeability water-flow channel between injection and production wells during the high water cut period, which intensifies the heterogeneity of the reservoir, resulting in the inability to displace the remaining oil in the low permeability area, low water injection sweep coefficient, much remaining oil, and inefficient or ineffective injection water circulation [11,12,13]. In view of the above problems, researchers have carried out a large number of studies, among which the use of gel to plug high permeability areas can effectively improve reservoir heterogeneity, improve the sweep efficiency of injected water, increase oil production, and reduce the water cut significantly. Field tests have proven that gel flooding can improve the development effect of conglomerate reservoirs in the high water cut period and is an effective way to achieve stable oil production and improve oil recovery [14,15,16,17].

Gel flooding is a method in which a certain amount of a cross-linking agent is mixed in polyacrylamide solution to form a dense polyacrylamide network structure, which can effectively block high-permeability layers, improve the utilization degree of low-permeability layers, ultimately achieve the purpose of profile control, and improve oil recovery [18,19,20]. The key parameters of gel flooding are the gelation time, adsorption quantity, viscosity, breakthrough pressure, and the RF and RRF of the gel. Many researchers have investigated these key parameters using experiments and obtained good results [21,22,23,24,25,26,27,28]. Zhang et al. [21] found that higher 2-acrylamido-2-methylpropane sulfonic acid content resulted in a higher gelation time for three gel systems prepared with S00, S30, and S50 polymers. Li et al. [22] discovered that the gelation time of a sustained-release crosslinker/water-soluble thixotropic polymer gel system decreased with the increase in poly (ethyleneimine) concentration. Wei et al. [23] reflected on the adsorption of a polymer gel system by measuring the polymer concentration and identified that under the same permeability, the higher the polymer concentration, the greater the adsorption quantity and the higher the adsorption rate. Zheng et al. [24] revealed that a polymer gel composed of 4000 ppm field-applied hydrolyzed polyacrylamide (HPAM) and a 3000 ppm Cr^3+^ acetate cross-linker had low viscosity at the beginning, and then, this increased with the gelation time, and the viscosity reached 11,000 mPa·s after 3 days of gelation time. Wu et al. [25] detected that a thixotropic and high-strength gel had a large swelling ratio, high breakthrough pressure, and good plugging ability in a swelling experiment and a slim tube experiment. Liu et al. [26] showed in their study that the RF became larger with the increasing polymer concentration through a polymer gel flooding experiment in a single sand pack and found that the polymer gel had good resistance to washing, with an RRF greater than 4.5 and a plugging rate higher than 78% after 20 pore volume (PV) water flooding. However, it has to be mentioned that there were inevitably errors in the experiment, and these errors should also be considered when determining the gel flooding parameters.

Although the key parameters of gel flooding can be obtained through laboratory experiments, this is not sufficient for the optimization of gel flooding in actual reservoirs. A group of scholars proposed a method that could quickly simulate and predict the profile control performance of multi-layer reservoirs [29], which could determine the overall decision of profile control well optimization, performance prediction, and consumption optimization using the production data of oil and water wells. Nevertheless, the determination of characteristic parameters is random and has some limitations in practical application. Numerical simulation can be used for the production prediction [30,31], feasibility assessment [32], and scheme optimization [33,34], which is one of the common means of reservoir development optimization research and is also applicable to the gel flooding of conglomerate reservoirs. Many researchers have applied the numerical simulation method to the optimization of gel flooding in conglomerate reservoirs and have achieved some results [35,36]. Wu et al. [35] obtained the distribution and gradation of water flow channels through numerical reservoir simulation and designed the slug parameters of three enhanced oil recovery (EOR) models based on the results. The field comparison test results showed that model 2 was optimal. Wu et al. [36] clarified the distribution characteristics of remaining oil by carrying out history matching and designed and optimized a subsequent development adjustment plan. However, at present, there are two major problems in the numerical simulation of gel flooding in conglomerate reservoirs. One is that the characteristic parameters of numerical simulation of gel flooding in conglomerate reservoirs are mostly directly based on the data obtained from physical experiments, without further consideration of experimental errors, and then better history matching is achieved by adjusting other parameters. Second, when conducting history matching of gel flooding in conglomerate reservoirs, the adjustment parameters are relatively large and have strong randomness, resulting in great time consumption for history matching. Therefore, how to better apply the parameter data obtained from physical experiments to numerical simulation and how to efficiently adjust the parameters to speed up history matching are urgent problems to be solved for the optimization of gel flooding in conglomerate reservoirs.

To address the current challenges faced in the optimization of gel flooding in conglomerate reservoirs, this paper took a conglomerate reservoir of Xinjiang A Oilfield as the research target and proposed a new optimization method for gel flooding. Firstly, the gel flooding parameters were obtained through physical experiments. Then, the gel flooding experimental model was established according to the target reservoir, and a parameter sensitivity analysis was carried out to reduce the randomness of parameter adjustment. On the basis of the parameter sensitivity study, history matching of the gel flooding experiment was carried out to confirm the correctness of the parameter sensitivity analysis results and further clarify the key parameters. After that, a conglomerate reservoir model of the Xinjiang A Oilfield was established, and history matching was carried out. Finally, a gel flooding scheme was designed and optimized to determine the optimal parameters of gel flooding during the high water cut stage in a conglomerate reservoir of the Xinjiang A Oilfield so as to achieve the purpose of optimizing gel flooding and improving oil recovery. This study can provide a reference for the development and optimization of similar oilfields, help to improve the oil recovery of the oilfields, and ease the tense situation surrounding domestic crude oil supply.

## 2. Materials and Methods

### 2.1. Physical Experiment

#### 2.1.1. Materials

**Figure 1:**
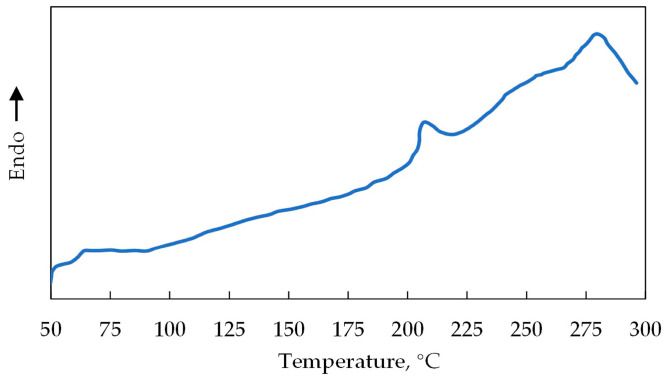
Differential scanning calorimetry (DSC) curve of the HPAM.

The polymer used in the experiment was hydrolyzed polyacrylamide (HPAM) with a molecular mass of 1.5 × 10^7^ g/mol and a hydrolysis degree of 26.36% from Beijing Hengju Chemical Group Corporation. Its glass transition temperature, melting point, and vaporized point are 188 °C, 209 °C, and 280 °C, respectively (Figure 1). The solid-state aldehyde cross-linking agent, as well as the accelerator, were the self-products of Beijing Hengju Chemical Group Corporation. The salinity of the simulated formation water was 13,456 mg/L, and its ionic component content is presented in Table 1. The crude oil sample was from the Xinjiang A Oilfield with a density and viscosity of 0.86 g/m^3^ and 15.80 mPa·s at 40 °C, respectively. The core samples were artificial cores; the #1–3 cores were used for the gel performance characterization experiment, and cores #4 and #5 were applied for the gel flooding experiment. Their basic information is provided in Table 2.

#### 2.1.2. Gel Preparation

Firstly, 1 L of HPAM solution with a concentration of 2500 mg/L was prepared with the simulated formation water; then 2 g of a cross-linking agent was added, and the solution was stirred in a JJ-1B stirrer from Xinrui Instrument Factory (Changzhou, China) at a speed of 400 revolutions per minute (rpm) for 2 h; after that, 0.3 g of the accelerator was added and stirred well to obtain a gel solution with a gel concentration of 4800 mg/L (recorded as $C_{1}$).

#### 2.1.3. Characterization of Gel Performance

**Figure 2:**
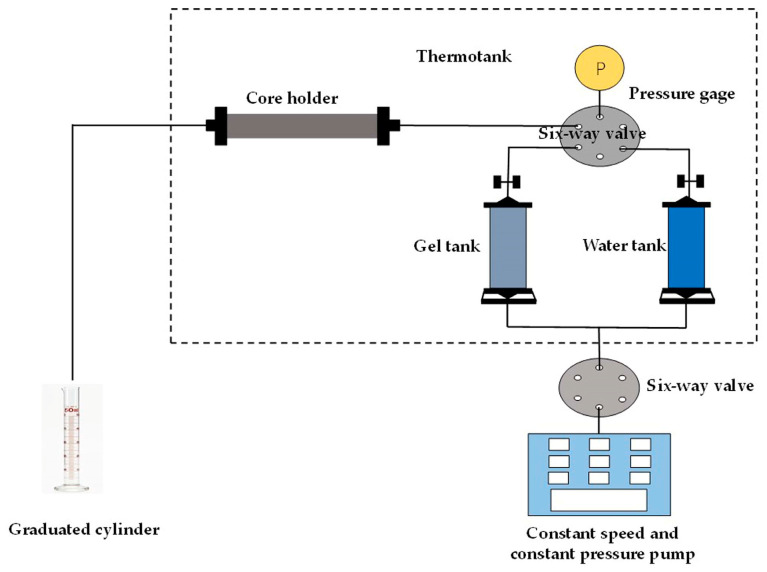
Schematic diagram of the gel performance characterization experimental device.

(1) The freshly prepared gel solution was sealed and placed in a constant-temperature water bath at 40 °C for static gelation. The viscosity was measured using a DV2T viscometer from Brookfield Engineering Laboratories, INC (Middleboro, MA, USA), and a viscosity versus time curve was plotted. The time when the viscosity inflection point occurred was the gelation time of the gel, and the viscosity when it tended to be smooth was the viscosity of the gel.

(2) The simulated formation water and the core saturated with the simulated formation water were loaded into the simulated formation water tank and core holder, respectively. The relevant experimental device was connected, as shown in Figure 2; the air in the device was completely evacuated, and the whole device was placed in a constant temperature thermotank at 40 °C for 12 h.

(3) Water flooding was carried out at a displacement rate of 0.2 mL/min, and the displacement pressure difference $\Delta P_{w1}$ was measured when it was stable.

(4) The freshly prepared 40 °C gel solution was filled into the gel tank, and the air in the device was completely evacuated. Gel flooding was conducted at a displacement rate of 0.2 mL/min. After injection of the 2 PV (recorded as $V_{1}$) gel solution, the displacement pressure difference $\Delta P_{i}$ was measured when it was stable. Then, gel flooding was stopped, and this condition was kept for 24 h.

(5) Water flooding was conducted at a displacement rate of 0.001 mL/min; the displacement pressure difference was recorded when the liquid at the outlet started to move, which is the breakthrough pressure of the gel. Then, the displacement rate was gradually increased to 0.2 mL/min. After the outlet was full of water, the displacement pressure difference $\Delta P_{w2}$ was measured after it was stable. 

(6) The total production liquid $V_{2}$ after gel injection and its gel concentration $C_{2}$ were measured. 

(7) The equations for the RF, RRF, and maximum adsorption quantity R are Equations (1)–(3), respectively.

(1) $$RF=\frac{\Delta P_{i}}{\Delta P_{w1}}$$

(2) $$RRF=\frac{\Delta P_{w2}}{\Delta P_{w1}}$$

(3) $$R=\frac{C_{1}V_{1}-C_{2}V_{2}}{W}$$

where W represents the weight of the core.

#### 2.1.4. Gel Flooding Experiment

**Figure 3:**
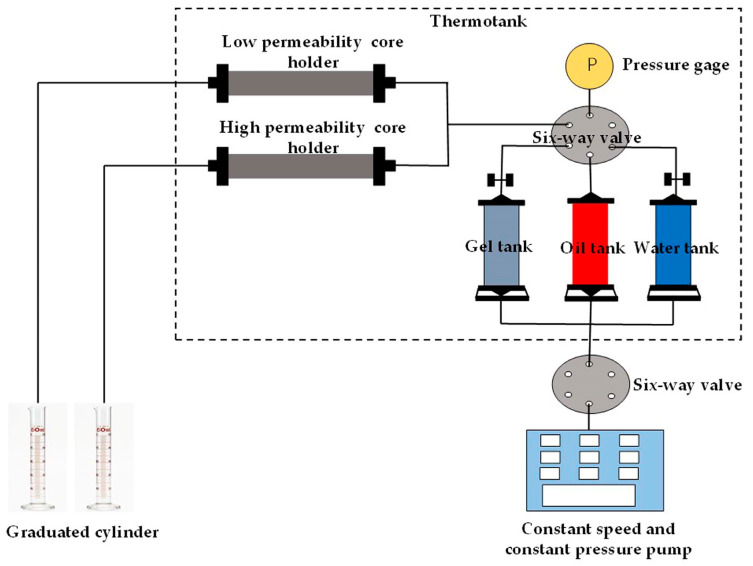
Schematic diagram of the gel flooding experimental device.

(1) The relevant experimental device was connected, as displayed in Figure 3. The simulated formation water, the core samples saturated with simulated formation water, and the crude oil samples were loaded into the simulated formation water tank, core holders, and crude oil tank, respectively. The temperature was set to 40 °C, and this condition was kept for 12 h.

(2) Saturating of the core samples with the crude oil sample was conducted, and irreducible water saturation was obtained. The specific process can be seen in [37,38]. After that, the condition was maintained for 12 h.

(3) The initial water flooding process was carried out at a displacement rate of 0.2 mL/min. The pressure at the inlet and the water and oil production at the outlet were recorded every 10 min until the injection of water reached 11.8 PV.

(4) A freshly prepared 40 °C gel solution was filled into the gel tank, and the air in the device was completely evacuated. Gel flooding was conducted at a displacement rate of 0.2 mL/min. The pressure at the inlet and the water and oil production at the outlet were recorded every 10 min until the injection of gel reached 0.5 PV. Then, gel flooding was stopped, and this condition was maintained for 24 h.

(5) The extended water flooding process was carried out at a displacement rate of 0.2 mL/min. The pressure at the inlet and the water and oil production at the outlet were recorded every 10 min until the injection of water reached 19 PV.

### 2.2. Numerical Simulation

Firstly, the STARS module in the CMG numerical reservoir simulation software version 2019.10 of Computer Modelling Group Ltd. was applied to establish the gel flooding experimental model. Then, considering that gel flooding history matching involved many parameters and took a considerable amount of time in order to improve the follow-up history matching speed of the gel flooding experiment and the conglomerate reservoir in the Xinjiang A Oilfield, the CMOST module in CMG software was used to conduct sensitivity analysis on the maximum adsorption quantity of the polymer, RRF and other parameters to determine the influence degree of each parameter, so that the parameter adjustment in the history matching process had a clear target. On the basis of parameter sensitivity analysis, history matching of the gel flooding experiment was carried out to confirm the correctness of the parameter sensitivity analysis results and further clarify key parameters to provide support for accelerating the subsequent history matching of the conglomerate reservoir in the Xinjiang A Oilfield. Based on the parameter sensitivity analysis and gel flooding experiment history matching, a conglomerate reservoir model of the Xinjiang A Oilfield was established, and history matching was conducted. Finally, based on the history matching of the conglomerate reservoir in the Xinjiang A Oilfield, the gel flooding scheme was designed and optimized to determine the optimal parameters of gel flooding at the high water cut stage so as to achieve the optimization of gel flooding in the conglomerate reservoir of the Xinjiang A Oilfield at the high water cut stage and improve oil recovery.

## 3. Results and Discussion

### 3.1. Experimental Results

#### 3.1.1. The Gelation Time and Viscosity of the Gel

**Figure 4:**
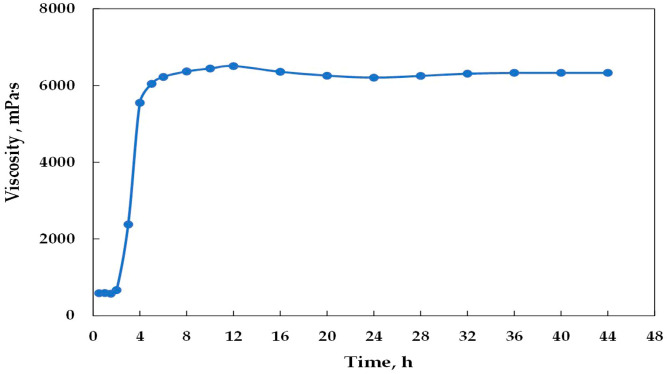
The change curve of gel viscosity with time.

The change curve of gel viscosity with time is illustrated in Figure 4. The gel viscosity began to increase significantly at about 2 h, which was due to cross-linking in the gel system, and gel viscosity became stable at 4 h. Therefore, the gelation time and viscosity of the gel were 4 h and 6332 mPa·s, respectively.

#### 3.1.2. Characteristic Parameters of Gel Flooding

The characteristic parameters of gel flooding were obtained through the characterization of gel performance, as shown in Table 3. The breakthrough pressure decreased as the permeability increased; this is due to the fact that the greater the permeability, the less resistance to fluid flow and the lower the breakthrough pressure [39]. The higher permeability resulted in lower RF and RRF; the main reason for this is that the retention of gel in the core with high permeability is reduced, which makes the flow of gel easier; the resistance declines, and the RF and RRF decrease [40].

#### 3.1.3. Experimental Analysis of Gel Flooding

**Figure 5:**
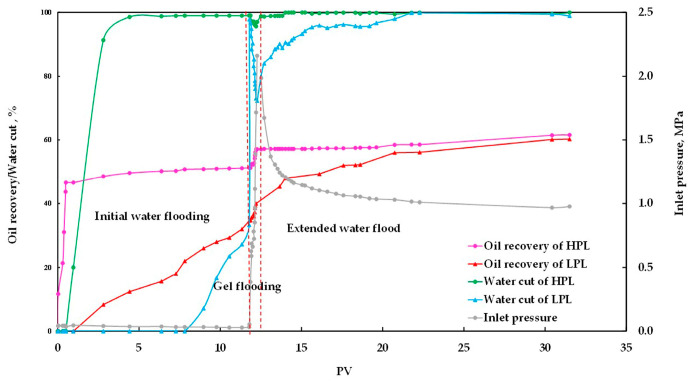
The production index curve of the gel flooding experiment.

The results of the gel flooding experiment are shown in Figure 5 and Table 4. During the initial water flooding process, the water cut and oil recovery of the high permeability core (called HPL) increased faster than that of the low permeability core (called LPL). After 2.8 PV water injection, the water cut and oil recovery of the HPL reached 91.30% and 48.54%, respectively, while those of the LPL were only 0 and 9.30%, respectively. After the initial water flooding process, the water cut and oil recovery of the HPL reached 99% and 51.26%, respectively, while those of the LPL were only 97.80% and 34.67%, respectively. This is due to the fact that the HPL makes it easier for water to flow, creating a dominant channel. The injection of 0.5 PV gel resulted in a minimum water cut of 96.97% and an EOR of 5.83% in the HPL, a minimum water cut of 72.20% and an EOR of 6.67% in the LPL. After gelation, the HPL was effectively blocked. The EOR of extended water flooding in the HPL was only 4.47%, while the EOR of extended water flooding in the LPL was as high as 18.93%, which is more than four times greater than that of the former. The ultimate oil recovery values of the HPL, the LPL, and the total were 61.56%, 60.27%, and 61.05%, respectively, and the efficacy of the gel flooding was efficient and well-demonstrated.

### 3.2. Numerical Simulation Results

#### 3.2.1. Gel Flooding Experimental Model

**Figure 6:**
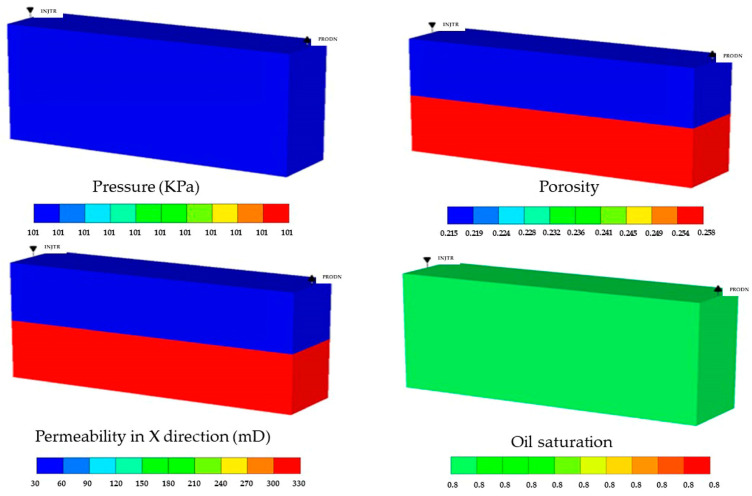
The distribution of initial pressure, porosity, permeability, and oil saturation in the gel flooding experimental model.

The grid number of the gel flooding experimental model was 100 × 1 × 2 = 200, and the grid size was 0.1 cm × 2.55 cm × 2.55 cm. The distribution of initial pressure, porosity, permeability, and oil saturation is provided in Figure 6.

#### 3.2.2. Sensitivity Analysis of Characteristic Parameters

**Figure 7:**
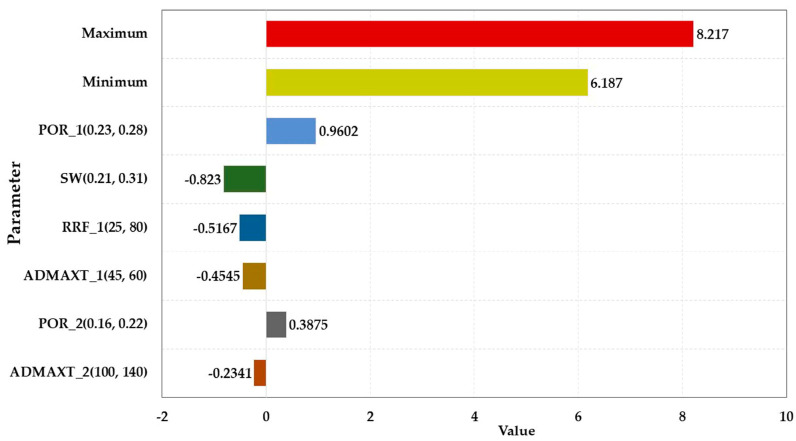
Parameter sensitivity analysis of the COP of the HPL.

**Figure 8:**
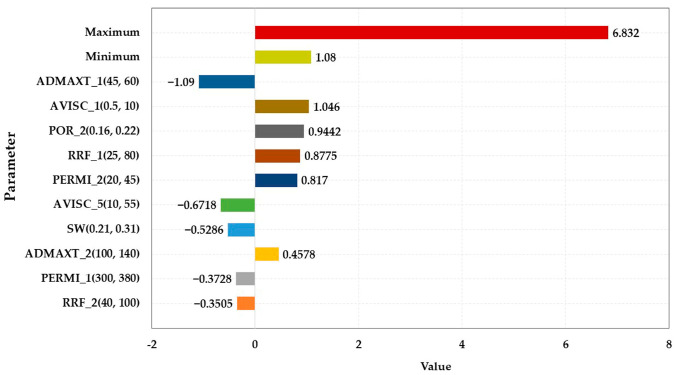
Parameter sensitivity analysis of the COP of the LPL.

**Figure 9:**
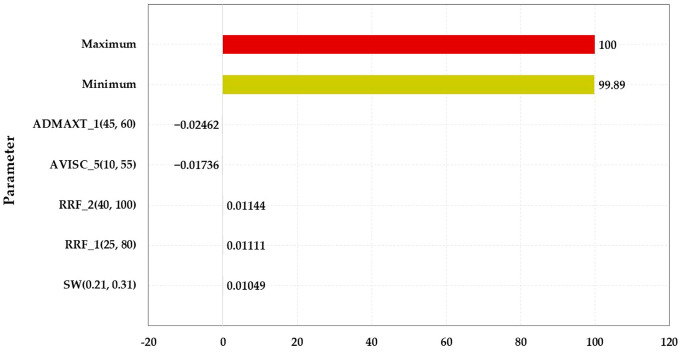
Parameter sensitivity analysis of the water cut of the HPL.

**Figure 10:**
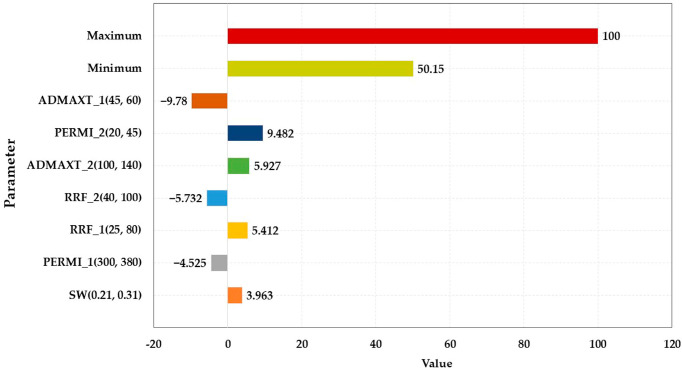
Parameter sensitivity analysis of the water cut of the LPL.

The main sensitivity analysis parameters are displayed in Table 5, and the sensitivity analysis results are illustrated in Figure 7, Figure 8, Figure 9 and Figure 10. The influence degree of the COP of the HPL from large to small was the porosity of the HPL, the irreducible water saturation, the RRF of the HPL, the maximum adsorption quantity of the HPL, the porosity of the LPL, and the maximum adsorption quantity of the LPL. That of the LPL from large to small was the maximum adsorption quantity of the HPL, the viscosity of the water, the porosity of the LPL, the RRF of the HPL, and the permeability of LPL. The maximum adsorption quantity of the HPL had a greater impact on the water cut of the HPL than the viscosity of the residual oil. The effect degree of the water cut of the LPL from large to small was the maximum adsorption quantity of the HPL, the permeability of the LPL, the maximum adsorption quantity of the LPL, the RRF of the LPL, the RRF of the HPL, the permeability of the HPL, and the irreducible water saturation.

#### 3.2.3. History Matching of the Gel Flooding Experiment

**Figure 11:**
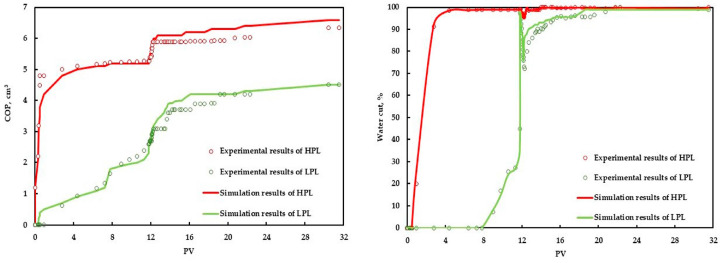
History matching results of the gel flooding experiment.

According to the sensitivity analysis results of the characteristic parameters, the influence of each parameter on the two important production parameters, the COP and the water cut of gel flooding, was clarified. Therefore, the history matching of the gel flooding experiment was effectively accelerated by modifying the main influencing parameters within a reasonable adjustment range for the production parameters that were not ideal, and good history matching was achieved, as shown in Figure 11.

#### 3.2.4. Model and History Matching of the Conglomerate Reservoir in the Xinjiang A Oilfield

**Figure 12:**
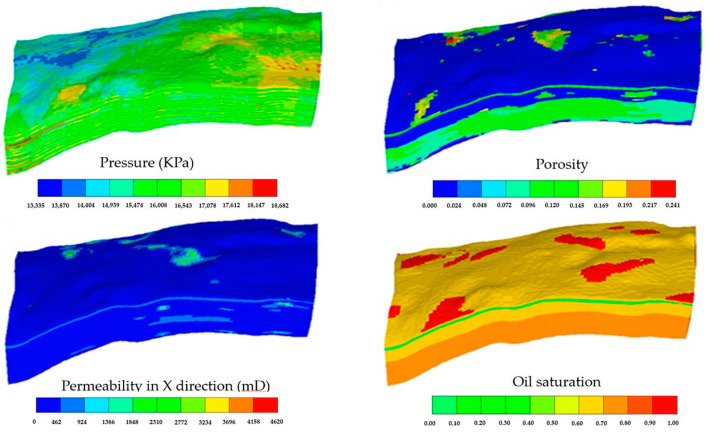
The distribution of initial pressure, porosity, permeability, and oil saturation in the model of the conglomerate reservoir in the Xinjiang A Oilfield.

The grid number of the model of the conglomerate reservoir in the Xinjiang A Oilfield is 87 × 65 × 35 = 197,925. The distribution of permeability, initial pressure, porosity, and initial oil saturation is shown in Figure 12.

The original geological reserve was 235 × 10^4^ m^3^, and the geological reserve in the model is 230 × 10^4^ m^3^, with an error of less than 2%. The reserve matching results were good.

**Figure 13:**
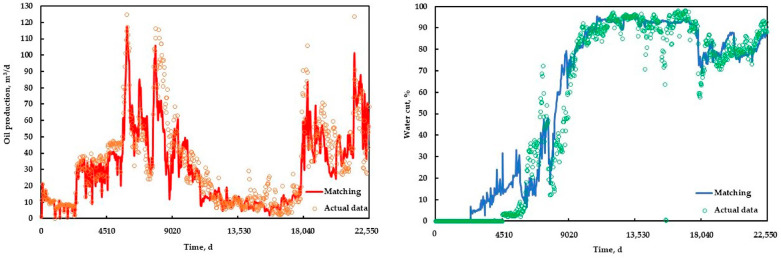
The history matching results of the reservoir.

**Figure 14:**
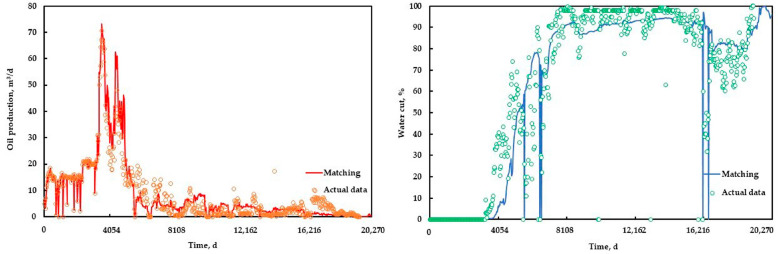
The history matching results of the typical single well.

Using the history matching of the gel flooding experiment as a reference, the history matching of the conglomerate reservoir in the Xinjiang A Oilfield was carried out based on the sensitivity analysis of characteristic parameters; its efficiency was also greatly improved, and a good history matching result was obtained. The history matching results of the reservoir and the typical single well are shown in Figure 13 and Figure 14, respectively.

#### 3.2.5. Scheme Design and Optimization

**Figure 15:**
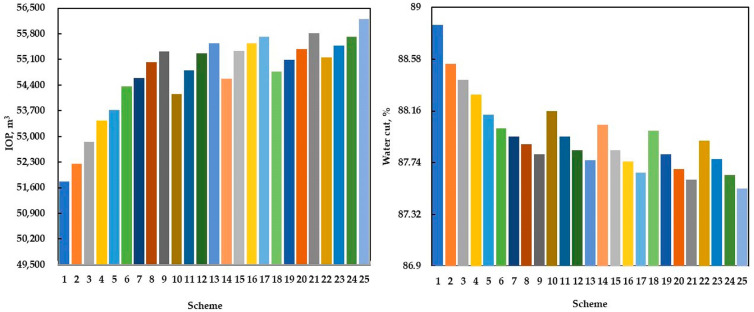
The results of different gel flooding schemes.

To determine the optimal gel injection volume, injection rate, and polymer concentration, five values were considered for each parameter, as displayed in Table 6. Different gel flooding schemes were designed by optimizing the combination of parameters, as shown in Table 7. In the scheme, the oil wells maintained the original fluid production rate; the injection production ratio was 1:1, and the prediction time was two years. The results of different gel flooding schemes are shown in Figure 15. The incremental oil production (IOP) in Figure 15 was the difference between the COP predicted by gel flooding and that predicted by water flooding.

**Figure 16:**
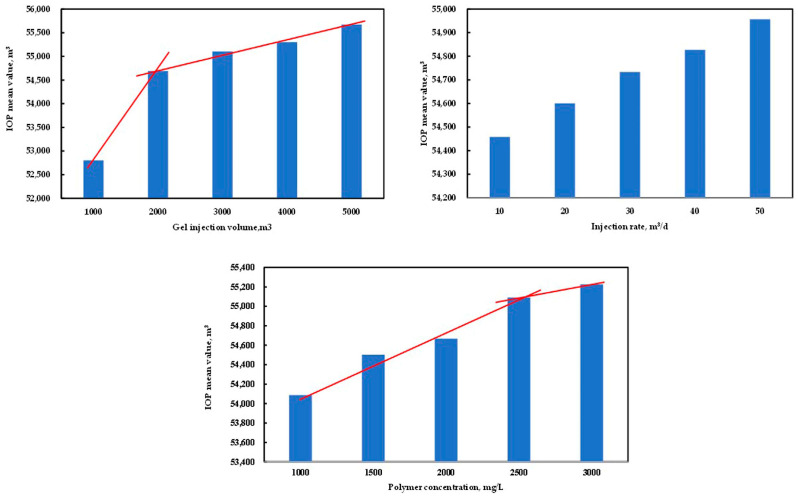
The IOP mean value of the gel injection volume, the injection rate, and the polymer concentration.

**Figure 17:**
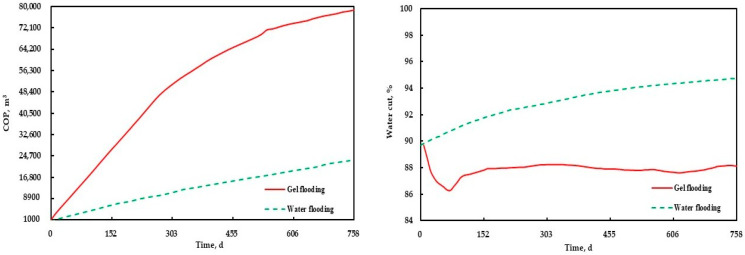
The COP and water cut prediction comparison between the optimal gel flooding scheme and water flooding.

**Figure 18:**
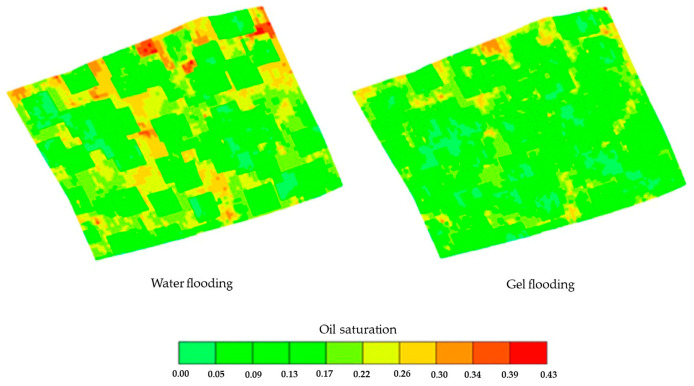
The remaining oil saturation distribution comparison between the optimal gel flooding scheme and water flooding.

As an important production index, the IOP was used for range analysis to determine the impact degree of different parameters and the best parameter value. According to Table 8, the IOP mean value of 1 corresponding to the gel injection volume was the average IOP of all the schemes with a gel injection volume of 1000 m^3^, and so on. The IOP mean value of different parameters and its range is shown in Table 8 and Figure 16. From Table 8, it can be seen that the impact on the gel flooding IOP from large to small was the gel injection volume, the polymer concentration, and the injection rate. From Figure 16, it can be determined that the optimal parameters of gel flooding in the conglomerate reservoir of the Xinjiang A Oilfield at the high water cut stage were as follows: the gel injection volume, injection rate, and polymer concentration were 2000 m^3^, 50 m^3^/d, and 2500 mg/L, respectively. The COP and water cut prediction comparison and the remaining oil saturation distribution comparison between the optimal gel flooding scheme and water flooding are provided in Figure 17 and Figure 18, respectively. Compared with water flooding, the water cut of the optimal gel flooding scheme decreased by 6.90%, and the oil recovery increased by 2.44%, showing a good effect by the EOR.

## 4. Conclusions

For a conglomerate reservoir, it is easy for a dominant water-flow channel to form during the high water cut period, but gel flooding is an effective method to solve this problem. However, the optimal design speed of gel flooding is affected by many factors, including numerous parameters, some errors in the parameters measured in this experiment, and no purposeful parameter adjustment in the process of numerical simulation. In this paper, a new optimization method of gel flooding was proposed. First, the characteristic parameters of gel flooding were measured through physical experiments; then, an experimental model of gel flooding was established, and parameter sensitivity analysis was carried out. Based on the parameter sensitivity analysis, the gel flooding experiment and actual reservoir history matching were conducted. With a clear aim, the speed of history matching was greatly improved, thus improving the optimization speed of gel flooding. This method was successfully applied to the conglomerate reservoir of the Xinjiang A Oilfield at the high water cut stage, and its optimal gel flooding parameters were obtained: the gel injection volume, injection rate, and polymer concentration were 2000 m^3^, 50 m^3^/d, and 2500 mg/L, respectively. Compared with water flooding, it was predicted that the water cut of the optimal gel flooding scheme would decrease by 6.90% and the oil recovery would increase by 2.44% in two years. The study of gel flooding in the conglomerate reservoir at the high water cut stage in this paper can provide a reference for similar oilfield development and has important significance in improving the application effect of gel flooding and EOR.

## Figures and Tables

**Table 1:** The ionic component content of the simulated formation water.

Ionic Component	Content, mg/L
HCO_3_^−^	1412.15
Cl^−^	5208.20
SO_4_^2−^	107.65
Na^+^	6412.20
Ca^2+^	208.15
Mg^2+^	107.65

**Table 2:** Basic information of the core samples.

Core No.	Length, cm	Diameter, cm	Porosity, %	Weight, g	Permeability, mD
#1	10.00	2.55	19.18	96.24	30
#2	10.01	2.53	22.35	95.33	170
#3	10.12	2.54	25.80	94.56	342
#4	10.07	2.54	26.98	94.25	345
#5	9.95	2.53	20.50	95.97	35

**Table 3:** Characteristic parameters of gel flooding.

Core No.	Maximum Adsorption Quantity, mg/g	Breakthrough Pressure, MPa	RF	RRF
#1	129.18	0.011	178.03	87.04
#2	71.46	0.006	120.76	69.81
#3	57.53	0.0043	81.50	27.60

**Table 4:** The statistics of the production indexes of the gel flooding experiment.

Core No.	Irreducible Water Saturation, %	Initial Water Flooding Oil Recovery, %	EOR of Gel Flooding, %	EOR of Extended Water Flooding, %	Ultimate Oil Recovery, %
#4	21.36	51.26	5.83	4.47	61.56
#5	30.13	34.67	6.67	18.93	60.27
Total		44.65	6.17	10.23	61.05

**Table 5:** The main sensitivity analysis parameters.

Name	Porosity	Permeability	Irreducible Water Saturation	Viscosity	Maximum Adsorption Quantity	RRF
Parameter	POR	PERMI	SW	AVISC	ADMAXT	RRFT

Note: Suffix 1 is the parameter of the HPL; suffix 2 is the parameter of the LPL, and suffixes 1–5 of the AVISC are the viscosity of the water, the crosslinking agent, the polymer, the gel, and the residual oil, respectively.

**Table 6:** Parameter design of gel flooding.

Parameter	Gel injection Volume, m^3^	Injection Rate, m^3^/d	Polymer Concentration, mg/L
Level 1	1000	10	1000
Level 2	2000	20	1500
Level 3	3000	30	2000
Level 4	4000	40	2500
Level 5	5000	50	3000

**Table 7:** The designed gel flooding schemes.

Scheme NO.	Gel Injection Volume, m^3^	Injection Rate, m^3^/d	Polymer Concentration, mg/L
1	1000	10	1000
2	1000	20	1500
3	1000	30	2000
4	1000	40	2500
5	1000	50	3000
6	2000	10	1500
7	2000	20	2000
8	2000	30	2500
9	2000	40	3000
10	2000	50	1000
11	3000	10	2000
12	3000	20	2500
13	3000	30	3000
14	3000	40	1000
15	3000	50	1500
16	4000	10	2500
17	4000	20	3000
18	4000	30	1000
19	4000	40	1500
20	4000	50	2000
21	5000	10	3000
22	5000	20	1000
23	5000	30	1500
24	5000	40	2000
25	5000	50	2500

**Table 8:** The IOP mean value of different parameters and its range.

Parameter	Gel Injection Volume, m^3^	Injection Rate, m^3^/d	Polymer Concentration, mg/L
IOP mean value 1	52,804.79	54,458.26	54,087.50
IOP mean value 2	54,691.17	54,600.09	54,502.43
IOP mean value 3	55,105.66	54,733.14	54,667.31
IOP mean value 4	55,300.76	54,827.55	55,092.85
IOP mean value 5	55,673.86	54,957.21	55,226.16
Range	2869.07	498.95	1138.66

## Data Availability

No new data were created.

## Abbreviations

RF, resistance factor; RRF, residual resistance factor; HPL, high permeability layer; LPL, low permeability layer; DSC, differential scanning calorimetry; COP, cumulative oil production; HPAM, hydrolyzed polyacrylamide; PV, pore volume; EOR, enhanced oil recovery; IOP, incremental oil production.
